# Supplementary material for: Composition and diversity of meibum microbiota in meibomian gland dysfunction and the correlation with tear cytokine levels
Source: PLoS One. 2023 Dec 22;18(12):e0296296. doi: 10.1371/journal.pone.0296296 (PMC10745150; doi:10.1371/journal.pone.0296296)
Supplement: S1 File — (DOCX) [file pone.0296296.s001.docx]

**Supplementary file 1.** MGD severity grading scale

| Severity | Stage | Symptoms | Clinical signs | Meibum quality* | Meibum expressibility** | Oxford grading system |
| --- | --- | --- | --- | --- | --- | --- |
| Mild | 1 | No discomfort, itching, or photophobia | Based on gland expression | 2–3 | 1 | No staining |
| Mild | 2 | Mild symptoms of ocular discomfort, itching, or  photophobia | Scattered lid margin features | 4–7 | 1 | 0– 3 |
| Moderate | 3 | Moderate symptoms of ocular discomfort, itching, or photophobia with limitations of activities | Lid margin vascularity,  plugged meibomian orifice | 8–12 | 2 | 4– 10 |
| Severe | 4 | Marked symptoms of ocular discomfort, itching, or photophobia with definite limitation of activities | Displacement of the mucocutaneous junction | ≥ 13 | 3 | 11–15 |

* Meibum quality is assessed at eight glands in central third of lower eyelid; grade 0 = clear, grade 1 = cloudy, grade 2 = cloudy with granular debris, grade 3 = thick, like toothpaste

** Meibum expressibility is assessed at five glands in central third of lower eyelid; grade 0 = all, grade 1 = 3–4, grade 2 = 1–2, grade 3 = 0

MGD, Meibomian gland dysfunction
