# Supplementary material for: Composition and diversity of meibum microbiota in meibomian gland dysfunction and the correlation with tear cytokine levels
Source: PLoS One. 2023 Dec 22;18(12):e0296296. doi: 10.1371/journal.pone.0296296 (PMC10745150; doi:10.1371/journal.pone.0296296)
Supplement: Attachment — Submitted filename: Response to Reviewers.doc [file pone.0296296.s003.doc]

[13 Nov 2023]

Kofi Asiedu
Academic Editor

*PLOS One*

Dear Editor:

We thank the reviewers for their insightful comments on the manuscript and have made revisions to the manuscript to address their concerns. Our responses to each of the comments and suggestions by the reviewers are listed below.

Sincerely,

Ngamjit Kasetsuwan

Department of Ophthalmology, Faculty of Medicine, Chulalongkorn University, Bangkok, 10330, Thailand

Excellence Center of Cornea and Limbal Stem Cell Transplantation, Department of Ophthalmology, King Chulalongkorn Memorial Hospital and Faculty of Medicine, Chulalongkorn University, Bangkok, 10330, Thailand

1873 Rama IV Rd, Pathum Wan, Bangkok, 10330, Thailand

[ngamjitk@gmail.com](mailto:ngamjitk@gmail.com)

Reviewers' comments:

Reviewer's Responses to Questions

**Comments to the Author**

1. If the authors have adequately addressed your comments raised in a previous round of review and you feel that this manuscript is now acceptable for publication, you may indicate that here to bypass the “Comments to the Author” section, enter your conflict of interest statement in the “Confidential to Editor” section, and submit your "Accept" recommendation.

Reviewer #1: (No Response)

Reviewer #3: All comments have been addressed

2. Is the manuscript technically sound, and do the data support the conclusions?

The manuscript must describe a technically sound piece of scientific research with data that supports the conclusions. Experiments must have been conducted rigorously, with appropriate controls, replication, and sample sizes. The conclusions must be drawn appropriately based on the data presented.

Reviewer #1: (No Response)

Reviewer #3: Yes

3. Has the statistical analysis been performed appropriately and rigorously?

Reviewer #1: (No Response)

Reviewer #3: Yes

4. Have the authors made all data underlying the findings in their manuscript fully available?

The [PLOS Data policy](http://www.plosone.org/static/policies.action#sharing) requires authors to make all data underlying the findings described in their manuscript fully available without restriction, with rare exception (please refer to the Data Availability Statement in the manuscript PDF file). The data should be provided as part of the manuscript or its supporting information, or deposited to a public repository. For example, in addition to summary statistics, the data points behind means, medians and variance measures should be available. If there are restrictions on publicly sharing data—e.g. participant privacy or use of data from a third party—those must be specified.

Reviewer #1: (No Response)

Reviewer #3: Yes

5. Is the manuscript presented in an intelligible fashion and written in standard English?

PLOS ONE does not copyedit accepted manuscripts, so the language in submitted articles must be clear, correct, and unambiguous. Any typographical or grammatical errors should be corrected at revision, so please note any specific errors here.

Reviewer #1: (No Response)

Reviewer #3: Yes

6. Review Comments to the Author

Please use the space provided to explain your answers to the questions above. You may also include additional comments for the author, including concerns about dual publication, research ethics, or publication ethics. (Please upload your review as an attachment if it exceeds 20,000 characters)

Reviewer #1: I have no additional comments for the authors. This manuscript had been revised according my comments ,and can be accept.

Reviewer #3: Page 6, lines 133-134: Please clarify how the meibomian gland orifices can be avoided when disinfecting eyelid margins. It does not sound that it can be promised. I suggest removing this sentence but adding the Reviewer’s comment in the limitation.

--------------------------------------------------------------------------------------------------------------------

*We have removed the sentence “The disinfection of the eyelid margin was carefully done while avoiding the area of meibomian gland orifices.” (page 6, line 124-126)*

*We also add the limitations of our study “Second, while disinfection of lid margin helps prevent contamination of meibum microbiota with lid margin microbiota, this process might have an effect on the resident bacteria in meibomian gland orifices.” (page 21, line 418-420)*

--------------------------------------------------------------------------------------------------------------------

Table 1. Please revise the following points:
- Spell out MGD in the table title
- Correct the misspelled word “particapants”,
- right and left eyes: please exchange (eyes) with (number).
- Please remove (number of yes) after lid assessment and add (present, number) next to each parameter

--------------------------------------------------------------------------------------------------------------------

*We have made correction in table 1., page 9-10)*

--------------------------------------------------------------------------------------------------------------------

Page 11, line 221-223: Please remove “for the severe MGD, moderate MGD, and HC groups” as it is redundant and confusing. Clarify what is “no different from the HCs”. Decreased diversity in severe MGD than in HCs? Revise accordingly.

--------------------------------------------------------------------------------------------------------------------

*We have removed the phase “for the severe MGD, moderate MGD, and HC groups” (page 11, line 212-213)*

*We also revised the sentence “Simpson’s index showed significantly decreased α-diversity in patients with severe MGD than those with moderate MGD. (p = 0.045; Fig 1). However, there was no difference in α-diversity between patients with severe MGD and HCs and patients with moderate MGD and HCs (p > 0.05; Fig 1).” (page 11, line 213-217)*

--------------------------------------------------------------------------------------------------------------------

7. PLOS authors have the option to publish the peer review history of their article ([what does this mean?](https://journals.plos.org/plosone/s/editorial-and-peer-review-process#loc-peer-review-history)). If published, this will include your full peer review and any attached files.


If you choose “no”, your identity will remain anonymous but your review may still be made public.


**Do you want your identity to be public for this peer review?** For information about this choice, including consent withdrawal, please see our [Privacy Policy](https://www.plos.org/privacy-policy).

Reviewer #1: **Yes:** Xiuming Jin

Reviewer #3: **Yes:** Sezen Karakus
